# Health checks for autistic adults: study protocol for a cluster randomised controlled trial

**DOI:** 10.1186/s13063-024-08641-5

**Published:** 2024-12-31

**Authors:** Jeremy R. Parr, Helen Taylor, Colin Wilson, Clare Scarlett, Sarah Al-Asmori, Carole Buckley, Sally-Ann Cooper, Cristina Fernandez-Garcia, Tracy Finch, Rhianna Lees, Nicholas Lennox, Hannah Merrick, Sebastian Moss, Christina Nicolaidis, Malcolm Osbourne, Dora M. Raymaker, Tomos Robinson, Anna Urbanowicz, James M. S. Wason, Barry Ingham

**Affiliations:** 1 Population Health Sciences Institute, Newcastle University, Newcastle upon Tyne, UK; 2 Cumbria, Northumberland, Tyne and Wear NHS Foundation Trust, Morpeth, UK; 3 Great North Children’s Hospital, Newcastle upon Tyne Hospitals NHS Foundation Trust, Newcastle upon Tyne, UK; 4 NHS North East and North Cumbria Integrated Care Board, Newcastle upon Tyne, UK; 5 Royal College of General Practitioners, London, UK; 6 School of Health and Wellbeing, University of Glasgow, Glasgow, UK; 7 Department of Nursing, Midwifery and Health, Northumbria University, Newcastle upon Tyne, UK; 8 Queensland Centre for Intellectual and Developmental Disability, MRI-UQ, The University of Queensland, Brisbane, QLD Australia; 9 NHS Northumberland Clinical Commissioning Group, Morpeth, UK; 10 School of Social Work, Portland State University, Portland, OR USA; 11 Department of Medicine, Oregon Health and Science University, Portland, OR USA; 12 The Kayaks Support Group, South Shields, UK; 13 Regional Research Institute for Human Sciences, Portland State University, Portland, OR USA; 14 Australia Institute for Health Transformation, Determinants of Health, School of Health and Social Development, Faculty of Health, Deakin University, Geelong, VIC Australia

**Keywords:** Autism, Intervention, RCT, Health checks

## Abstract

**Background:**

Autistic people commonly have physical and mental health conditions. They also frequently experience barriers to accessing healthcare, contributing to problems identifying and treating health conditions. These factors may lead to increased and earlier morbidity and lower average life expectancy for autistic people. Health checks specifically designed for autistic people, incorporating adjustments to healthcare, may help to overcome these barriers and reduce health inequalities. This trial aims to investigate the clinical and cost-effectiveness of a primary care health check for autistic adults and explore factors related to implementation such as acceptability and feasibility of delivery. The trial is co-designed and delivered by health professionals, autistic people, carers and supporters, and researchers.

**Methods:**

This is a clinical and cost-effectiveness, cluster randomised controlled trial of a primary care health check for autistic adults. Primary care practices will be randomised into one of two groups (intervention or control). Two hundred autistic adults (aged 18 years and over) who provide baseline data will be recruited via participating practices. Data will be collected through quantitative and qualitative methods. The primary outcome will be the incidence of new health needs/conditions detected and met at 9 months (data gathered from participant’s GP records). Secondary outcomes will include the following: cost-effectiveness, measured as incremental cost per quality-adjusted life year gained over 9 months; the extent of health monitoring and health promotion needs met at 9 months; the incidence of social care needs identified at 9 months; changes in participant or carer general health; changes in quality of life; primary and secondary health and social care resource usage and costs. A qualitative study will explore views about the acceptability of the health check, its utility and future use.

**Discussion:**

This study will examine the effectiveness and cost-effectiveness of a primary care health check for autistic adults in identifying new health conditions and needs. If the intervention is effective, it would provide strong evidence for implementation into routine healthcare, therefore enabling earlier health condition diagnosis and opportunities for treatment, reducing the health inequalities experienced by autistic people.

**Trial registration:**

ISRCTN, retrospectively registered on 20 July 2023. https://www.isrctn.com/ISRCTN30156776 (ISRCTN registration number: 30156776).

**Supplementary Information:**

The online version contains supplementary material available at 10.1186/s13063-024-08641-5.

## Background

Autistic people^1^ experience more physical and mental health conditions than non-autistic people and premature mortality [1–5]. Several studies have highlighted a range of barriers faced by autistic adults in accessing healthcare services, which likely contribute to difficulties in identifying and treating autistic adults’ health conditions [6]. These include problems with patient-provider communication [7], a lack of autism training among healthcare providers [8], sensory overload due to healthcare environments [9], cognitively inaccessible systems and services, and a lack of adequate support [10, 11]. Health checks identify some important conditions that affect health and wellbeing and are available to some groups of people in several countries. Regular health checks designed for autistic people, incorporating adjustments to healthcare practices, may help to overcome these barriers and reduce health inequalities for autistic people.

Annual health checks for people with intellectual disabilities^2^ were developed and evaluated in several Randomised Controlled Trials (RCTs) [12, 13], leading to arrangements being put in place for people with intellectual disabilities to receive an annual health check within primary healthcare services. Recent studies found that health checks for people with intellectual disabilities consistently identified unmet health needs, resulted in targeted actions to address those needs, and reduced mortality [12–16]. However, these may not be available to or appropriate for autistic people who do not have an intellectual disability. The development and implementation of a health check specifically for autistic adults might bring similar benefits.

Approaches that are similar to a health check for autistic people have been developed in the US and Australia. AASPIRE (Academic-Autism Spectrum Partnership in Research and Education) developed the AASPIRE Healthcare toolkit [10] to support autistic people in accessing primary care and to support primary care health professionals (hereafter health professional) in meeting the health needs of autistic people. This includes the Autism Healthcare Accommodations Tool (AHAT), which creates a customised ‘accommodations report’ for individuals to share with their healthcare providers highlighting things that would make healthcare easier for the individual. Evaluation of the AHAT in the US found that it significantly reduced barriers to healthcare and increased ratings of patient-provider communication and patient healthcare self-efficacy [10]. In Australia, use of the AASPIRE Healthcare toolkit [10] was explored with six autistic adults who reported the toolkit facilitated their interactions with health professionals and that an adapted version specific for the Australian context may be beneficial [17]. Also in Australia, the Comprehensive Health Assessment Program (CHAP) was developed to provide a health check for people with intellectual disabilities [13]. This has subsequently been adapted for autistic adults and adolescents and its effectiveness is being evaluated.

Autistic people, supporters, and professionals have agreed that the creation and evaluation of a health check for autistic people is a priority to address healthcare access and the possible impact of healthcare barriers on wellbeing and life expectancy as research priorities [18]. Policy makers have also identified the development and evaluation of health checks for autistic people as a priority [19, 20]. To address this research priority, we designed a research programme with three stages: first, we identified the facilitators and barriers to healthcare access for autistic adults through group discussions and interviews with autistic people, supporters, and health professionals [6, 21]. Second, we created a health check for autistic people in partnership with autistic people, carers and supporters, and health professionals [21, 22]. This comprised primary care training, a health checklist, and a questionnaire (online and paper) to be completed by autistic people prior to the appointment and used to gather health-related information and the adjustments needed to make the appointment most acceptable. The final stage of the programme is the cluster randomised control trial (RCT), described below.

### Objectives

The aim of this cluster RCT is to evaluate the clinical and cost-effectiveness of a health check for autistic adults compared to usual care at 9 months follow-up. We will assess the clinical effectiveness of this intervention by measuring the incidence of new health needs and conditions detected and met at 9 months. There are a number of secondary outcomes including (i) the extent of health monitoring and health promotion needs being met at 9 months, (ii) changes in participant or carer-related general health and quality of life, (iii) the incidence of social needs identified at 9 months, (iv) changes in primary and secondary health and social care resource usage and costs, and (v) incremental cost per quality-adjusted life year (QALY). A qualitative study will also explore views about the acceptability of the health check, its utility and future use.

## Methods and design

### Study design

This study is a two-group cluster RCT non-blinded to participants but blind to the outcome assessors. Primary healthcare services (National Health Service general practices, referred to as ‘practices’ from hereon) will be randomised to either deliver the health check for autistic adults (intervention arm) or provide usual care (control arm), in keeping with previous research methods from published research, and following discussion with autistic people during the research design stage. We will offer adults aged 18 years or above with a clinical diagnosis of autism spectrum disorder at participating practices the opportunity to take part. We will administer assessments on entry to the trial (baseline) and at 3 months, 6 months, and 9 months after the health check, for the intervention arm, or after baseline measures are completed, for the usual care group. We will also gather qualitative data on the acceptability and feasibility of the health check to inform its future use. See Additional File 1 for Standard Protocol Items: Recommendations for Interventional Trials (SPIRIT) checklist for study.

### Study setting

We will invite practices from the geographical areas covered by the UK North East and North Cumbria, Yorkshire and Humber, North West Coast, and Greater Manchester National Institute for Health and Care Research (NIHR) Research Delivery Networks (RDNs) to take part in the trial. Practices will not be eligible to participate if they are currently offering health checks to autistic people or who have plans to do so in the near future.

### Recruitment and eligibility criteria

We will recruit two hundred autistic adults via participating practices: approximately 100 to each trial arm. Autistic adults are eligible if they meet the following criteria: (1) a clinically confirmed autism diagnosis and (2) aged 18 or over. Autistic adults are not eligible to take part if they meet one or more of the following criteria: (1) have had an NHS learning disability annual health check in the last 12 months or are anticipated to attend one in the next 3 months; (2) are unable to consent for themselves and do not have a relative or supporter who can consent for them, in keeping with England’s Mental Capacity Act; (3) people in the final stages of a terminal illness; (4) their GP considers it inappropriate to invite them.

### Participant identification and recruitment process

**Fig. 1:**
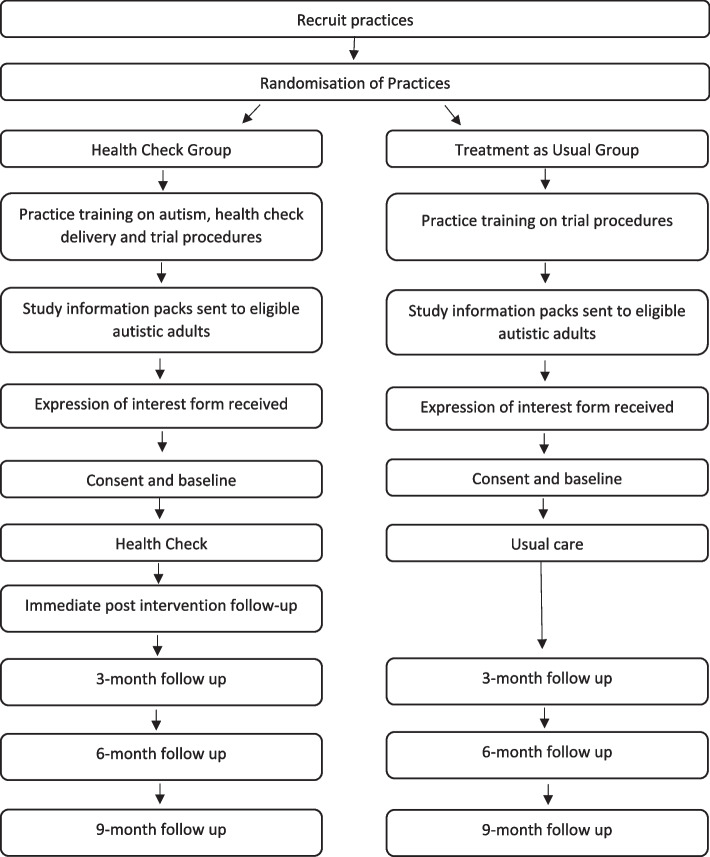
Health checks for autistic adults trial procedure

Participating practices will identify autistic adults to send a study information pack explaining the study and inviting participation. For those people unable to consent for themselves, the study information pack will be sent to a relative or carer (referred to as a ‘consultee’ in the Mental Capacity Act), asking them to advise whether the person should take part. The autistic adult or their consultee will then be asked to complete the measures from the perspective of the autistic adult. Easy read and short versions of all information sheets are available to possible participants. Interested autistic adults will complete an expression of interest form and send this to the research team. A member of the research team, RDN staff, or primary care staff will arrange a meeting with the prospective participant to discuss the trial in more detail and take informed consent using face to face, video, or telephone means, in line with the participant’s preference. See Appendix 1 for copies of the consent forms. After informed consent, the research team, RDN staff, or primary care staff will collect baseline data on participants. See Fig. 1 for the flow chart of trial procedure.

### Randomisation

Practices will be randomised to the intervention (health check) arm or the control (usual care) arm prior to participant recruitment via computer-generated sequence blocks [2, 4, 23]. Randomisation will be stratified by practice list size (small < 10,000 and large ≥ 10,000). A member of the research team independent of trial data collection will oversee this process and inform the practices of the randomisation outcome. The research team will make autistic participants aware as to whether they are in the health check or usual care arm in the study information pack. Due to the design of this trial, it is not possible for practices or participants to remain blinded to group.

### Health check for autistic adults intervention

Primary care staff in the health check arm will undertake training on autism and health check delivery provided by the research team. Training will comprise two mandatory modules (modules 1 and 2) and one optional module (module 3). Module 1 is an autism awareness e-learning programme for primary care staff that takes approximately 30 min to complete [24]; module 2 is a 1-h online session that builds on module 1 knowledge and outlines the core components of the health check, its delivery, and barriers to engagement. Module 3 comprises a number of continuing professional development sessions aiming to build enhanced knowledge and skills related to the delivery of healthcare to autistic people.

The Health Check for Autistic Adults is a biopsychosocial developmental health check designed to improve access and engagement with healthcare services for autistic adults, enable the identification of physical health, mental health, and social needs in autistic adults, and provide an opportunity for health promotion, health monitoring, and signposting autistic adults to services for support.

The Health Check for Autistic Adults comprises Primary Care staff training (as described above), a Health Check Pre-Appointment Questionnaire (Health Check PAQ) completed by the autistic person (or their supporter), and a health check appointment with a health professional.

Health Check PAQ: The practice will send autistic participants the Health Check PAQ (paper or online version as preferred by the participant) to complete and return to them before their health check appointment. This questionnaire comprises three parts: (1) questions about the autistic participant and their communication and sensory needs; (2) questions about what reasonable adjustments the autistic participant would like to help them engage with and attend the health check appointment; (3) questions about the autistic participant’s general health and wellbeing, including physical and mental health symptoms/conditions known or suspected to be more prevalent in autistic people and conditions that result in high rates of morbidity, or mortality. The online version of the questionnaire will automatically produce a summary report containing key information which a member of the primary care staff will upload to the autistic participant’s health record. Primary care staff will create and upload to the participant’s health record a summary report for autistic participants completing a paper version using the information provided.

Prior to the health check appointment, primary care staff will read the relevant parts of the completed Health Check PAQ Summary Report and will aim to implement reasonable adjustments as requested by the participant. Where practices are unable to put all the requested adjustments in place, primary care staff will contact the participant to discuss what adjustments they can offer, unless the participant indicated that they would not like to be contacted on the Health Check PAQ.

The practice will invite autistic participants to attend a health check appointment after their Health Check PAQ is received. Autistic participants will receive a leaflet containing details about the health check appointment. An appropriate health professional will complete the Health Check appointment, taking account of the information in the Health Check PAQ Summary Report. The Health Check appointment covers health promotion, health screening, vaccinations, physical health, mental health, daily functioning, relationships and sexual health, a brief medication review, and a physical examination. The health check appointment takes around 45 min to complete. Practices will offer flexibility around how the health check appointment is conducted (i.e. face-to-face, via video, via telephone or home visit) in line with the autistic participant’s preference and needs. The health professional will always undertake the physical examination in a face-to-face appointment. The health professional will communicate a Health Action Plan to the autistic participant at the appointment (with a written copy offered) based on the discussions, including highlighting the needs identified. They will also arrange any tests, interventions, or onward referrals as part of NHS care based on the Health Action Plan.

### Usual care

Participants in the usual care arm will receive primary healthcare services as usual. They may receive other health checks routinely delivered as part of usual care.

### Measures

**Fig. 2:**
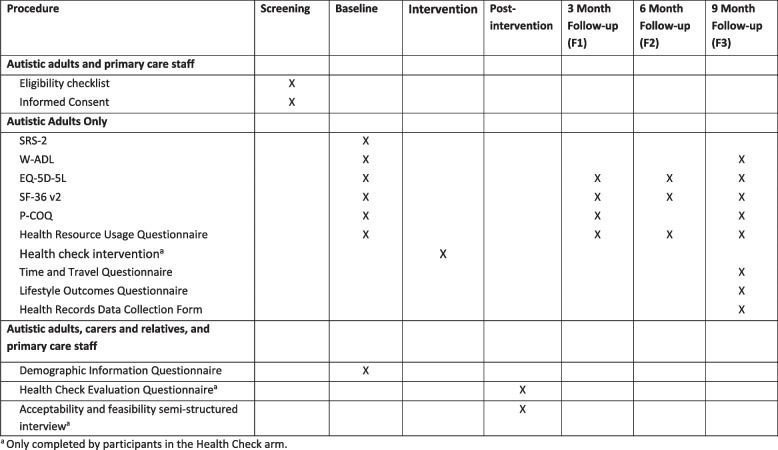
Intervention and data collection time points

See Fig. 2 for the time points at which data are collected.

#### Baseline characterisation

Following informed consent, participants will complete baseline measures (on paper or online via Qualtrics software [25]). These comprise a set of self-report measures to enable characterisation of the individual’s autism profile, general ability, and demographics. Participants can choose to complete the measures independently or with support from a carer or supporter or the person taking consent (researcher, RDN staff, or primary care staff). The following self-report measures will be collected:

- Demographics: we designed a bespoke demographic questionnaire for the study with versions for autistic adults, carers and supporters, health professionals, and administrate staff. The autistic adult’s version includes questions about personal characteristics, their living situation, employment and education, learning ability, support needs, and health conditions. For other participants, demographic information about personal characteristics is collected
- Social Responsiveness Scale – Second Edition (SRS-2) [26] is a 65-item standardised self-report questionnaire used extensively to rate the social communication features of autistic adults and children. Reported estimates of internal consistency between 0.94 and 0.96 and predictive validity of the adult form found a specificity level of 0.60 and sensitivity of 0.86 [27]
- Waisman Activities of Daily Living Scale (W-ADL) [28] is a measure of independence validated in people with a broad range of developmental disability diagnoses. Analysis indicates that it is reliable over time (*κ* = 0.92–0.93), with high levels of internal consistency across disability groups (Cronbach’s *α* = 0.88–0.94) and criterion validity when compared to the Vineland Screener Adaptive Behavior Composite Score (*r* = 0.78)

#### Outcome measures

The primary outcome for the trial will be the incidence of new health needs or conditions detected and met at 9 months. The primary analysis will report the mean number of identified needs that are met for each group. A number of secondary outcomes will also be evaluated including the following: the extent of health monitoring and health promotion needs met at 9 months, the incidence of social needs identified at 9 months, change in participant or carer general health, changes in quality of life, and changes in primary and secondary health and social care resource usage and costs. The primary economic outcome will be the incremental cost per QALY gained at 9 months.

Effectiveness measures:

- Bespoke Health Records Data Collection Form: we will collect health and social data routinely recorded by practices alongside information about previous health checks and health screening. This was informed by data collection from Cooper et al. [12] and includes the following: short and medium term health outcomes including number and type of new health needs/new conditions/new manifestation of an existing or previously diagnosed condition identified, any referrals to secondary care and/or general hospitals and results, screening or treatments undertaken (including management of Quality & Outcomes Framework qualifying long term conditions and review of medications or other treatment plans), and whether these health needs/conditions were resolved
- Primary Care Outcomes Questionnaire (P-COQ [29]) is a standardised 24-item questionnaire to measure outcomes in primary care. Analysis has identified 4 domains (health and wellbeing, health knowledge and self-care, confidence in health provision, and confidence in health plan) with high levels of internal consistency across each domain (Cronbach’s *α* = 0.77–0.95) and correlate with comparator Patient-Reported Outcome Measures (PROMs)
- Bespoke Lifestyle Outcomes Questionnaire gathers information about lifestyle choices and health related behaviours

Acceptability and feasibility measures:

- Bespoke Health Check Evaluation Questionnaire gathers information about the experience of completing/receiving the Health Check PAQ and attending/delivering the health check appointment. This questionnaire will only be completed by those in the health check arm immediately after the health check. There are parallel versions for autistic adults, carers and supporters, and health professionals
- Appointment acceptance and attendance rates: we will collect data on the acceptance of health check appointments in the health check arm. We will use a brief questionnaire to ask participants who did not attend an offered health check appointment why they did not attend

Digital tool measures:

- Information about the digital version of the Health Check PAQ, e.g. time taken to complete and whether completed in one visit: We will gather data using a Health Check Evaluation Questionnaire and semi-structured interviews post-health check

Health economic measures:

- EQ-5D-5L [30] [31] is a standardised measure of health-related quality of life allowing for the calculation of QALYs, used by NICE routinely to measure intervention effectiveness [32]. There is evidence that the measure demonstrates convergent validity with all items of the World Health Organisation Five Wellbeing Index (WHO-5 [33])
- Short-Form Health Survey version 2 (SF-36v2) [34]) is a standardised measure of health-related quality of life. The SF-36v2 assesses eight health concepts: limitations in physical activities because of health problems, limitations in social activities because of physical or emotional problems, limitations in usual role activities because of physical health problems, bodily pain, general mental health (psychological distress and wellbeing), limitations in usual role activities because of emotional problems, vitality (energy and fatigue), and general health perceptions. The SF-36v2 has high precision and has been extensively validated as a tool to measure health-related quality of life
- Bespoke Health Resource Usage Questionnaire: to capture data on primary and secondary healthcare resource usage, use of social services and prescribed medication
- Bespoke Time and travel costs questionnaire: to capture time and travel costs related to contacts with healthcare providers

A researcher, RDN staff, or primary care staff will collect baseline questionnaires. Follow-up measures will be collected by a researcher or RDN staff. We will allocate all participants a unique number that will identify them on all assessment forms throughout the trial. Questionnaires will be completed on paper or online (via Qualtrics software [25]) in line with participants’ preferences. To promote retention, we will update participants regularly on study progress via quarterly newsletters and participants will receive a £20 voucher after the 3-month follow-up and a £30 voucher after the 9-month follow-up. A researcher will input all paper data into a database, and this will be stored separately to identifying data. Data entry and validation will be a continuous process, with a proportion of double data entry of questionnaire data, so that we will identify and address problems immediately. We will carry out range checks of data values and manual checks of source data will be undertaken where required. Those completing online questionnaires using Qualtrics software will be sent a personalised link, linked to their unique ID number. A researcher will download online questionnaires onto secure servers and merge with the data from paper version for analysis. Supported by primary care and/or RDN staff, a non-blinded researcher will collect, anonymise, and securely store data collected from health records. This anonymised information will then be rated by members of the study team who are blind to trial arm allocation and who have not had contact with participants. We will manage missing questionnaire data at item level in accordance with the manual for each questionnaire. We will treat all study data in accordance with the latest directive on Good Clinical Practice (2005/28/EC). We will invite a sample of participants (autistic adults, carers and supporters, and primary care staff) to take part in an interview. A researcher will conduct and record semi-structured interviews face-to-face, via video, or telephone, in line with participants’ preferences. An external transcription agency will transcribe all interviews and a researcher will anonymise them.

### Internal pilot

As there had not been a feasibility and acceptability study before, we conducted a 6-month internal pilot in April to October 2023 to measure the rate of the following: recruitment to the health check and usual care arms, practice’s health check delivery, completion of measures, and retention. We compared the rates to pre-specified progression criteria: recruitment of ≥ 20 autistic people per trial arm = continue recruitment, recruitment ≥ 15 autistic people per trial arm = recruit additional practices and review after 3 months, recruitment of ≤ 10 autistic people = discuss options with funders. We also undertook a safety analysis once 20 health checks had been conducted (between March and July 2024). This focused on identifying any possible harms or adverse effects of undertaking the health check. The harms analysis will consider the frequency of potential harms recorded in the health records and those reported by autistic people, carers and supporters, and health professionals between the trial arms: distress or anxiety, depression or low mood, increased patient consultations, and other harms.

### Adverse event recording

Serious adverse events are not anticipated in the study; therefore, any event will be recorded as an unexpected adverse event. We will record the following events: a significant (as reported by treating health professional) increase in anxiety symptoms, a significant (as reported by treating health professional) increase in low mood or depressive symptoms, suicidal ideation, physical or emotional harm resulting from additional investigations or healthcare services received, unnecessary physical investigations, any safeguarding concerns, or any other significant event. The chief investigators will immediately review serious events in accordance with the trial protocol and respond in accordance with relevant NHS protocols. We will report all serious events to the sponsoring NHS Trust and the reviewing REC as per the standard operating procedure of the sponsoring NHS Trust.

Newcastle University and Cumbria, Northumberland, Tyne, and Wear Foundation Trust have indemnified the trial.

### Sample size justification

The sample size of 200 was chosen based on the number of participants that could be recruited given the funding envelope and time available. This number compares favourably with previous health checks trials that found a difference between groups [12]. Assuming an attrition rate of 10%, the sample size was inflated to 220 (110 per arm).

### Data analysis plan

#### Statistical analysis for quantitative analysis

We will carry out all statistical analyses in accordance with the intention-to-treat principle where all participants’ outcomes are analysed as randomised. We will report all participant flow in accordance with CONSORT statement for non-pharmacological interventions. A full statistical analysis plan will be written and signed-off before trial statisticians receive any unblinded data. The following represents a summary of the planned analyses.

The underlying structure of the data displays a two-level hierarchical structure, in which one level (participants) is nested within another (practices). Members of the same practice share contextual factors and GP conduct, meaning 2 people of the same practice are more likely to have similar data relative to two people from different practices. This clustering effect can be measured using the intra class correlation coefficient (ICC). To compare the groups for the primary outcome, taking into account the clustering effect, we will use a mixed-effects Poisson regression model that has fixed effects for cluster size, treatment allocation, age, and sex. If this model does not converge, we will reduce the number of fixed effects in the model and then (if it still does not converge) change the random cluster effect to a fixed effect.

We will conduct subgroup analyses for the primary outcome by including the subgroup variable and an interaction parameter between subgroup variable and intervention. The subgroups we will investigate are number of conditions at baseline (above median vs median or below) and presence vs absence of mental health condition at baseline.

For secondary outcomes, we will use appropriate mixed-effects regression models, depending on the outcome type. If the outcome is a questionnaire that is also assessed at baseline, the model will adjust for that baseline measure.

Participants who withdraw consent for the collection of routinely available data prior to month 9 will not be included in the primary outcome analysis. We do not anticipate there being missing data for the primary outcome as it is based on routinely collected data. Secondary analyses will be complete-case analyses. For outcomes that are significant, if there is substantial differential dropout between treatment groups, we will apply multiple imputation with at least 20 imputed datasets and undertake sensitivity analyses to explore the robustness of results to the missing not at random assumption. We will record and tabulate the number of serious adverse events per group.

#### Economic evaluation

The economic evaluation will involve a within-trial economic evaluation to estimate the incremental cost per QALY gained at 9 months. The analysis will take the perspective of the UK National Health Service (NHS) and Personal Social Services. We will consider a wider societal perspective by analysing the costs borne by the participants and their carers. For the within trial economic evaluation, QALYs will be estimated from responses to the EQ-5D-5L administered at baseline and 3, 6, and 9 months follow-up. As per the National Institute for Health and Care Excellence (NICE)’s current guidelines [35], the EQ-5D-5L results will be converted (‘cross-walked’) into EQ-5D-3L scores. These values will be used to estimate QALYs for each participant using the area under the curve approach [36]. In a sensitivity analysis, QALYs will also be estimated from responses to the SF-36v2 administered at the same time points. The responses to the SF-36v2 will be converted into SF-6D tariffs [37] and QALYs estimated using the same methodology as described above.

Intervention cost data: The cost of delivering the health check in primary care will be calculated using the information captured by the Health Check Evaluation Questionnaire. This includes data relating to the training required for delivering the health check and the time and resources for delivering the health check appointment and creating the health action plan.

Resource use data: The costs of all health services will be estimated using routine data sources, e.g. Unit Costs of Health & Social Care for primary and social care [38], NHS National Schedule of Reference Costs, and the British National Formulary for medications [39]. For each participant, we will combine measures of the use of resources with unit costs to provide a cost for that participant and mean cost at 9 months for each randomised group.

#### Quality of life data

The QALY will be used in the cost-effectiveness analysis as the health outcome as it is recommended by NICE. QALYs will be used to assess the health gain of participants over the 9-month trial period.

#### Cost-effectiveness analysis

The cost-effectiveness analysis will take the form of a cost-utility analysis and conducted under the intention-to-treat principle and will take the perspective of the healthcare provided (UK NHS). We will combine both costs and QALY data to estimate the incremental cost-per QALY, derived from responses to the EQ-5D-L questionnaire, gained for the comparison of each randomised group at 9 months. The cost-utility analysis will include deterministic and stochastic sensitivity analysis, presented as point estimates and cost-effectiveness acceptability curves (CEACs). We will apply techniques such as two-stage non-parametric bootstrapping alongside deterministic sensitivity analyses to minimise any uncertainty surrounding the incremental cost per QALY gained.

### Acceptability and feasibility of health check

Descriptive data from the health check evaluation questionnaires provided by autistic adults, carers and supporters, and health professionals will be summarised, and open text responses will be coded.

### Qualitative study of acceptability and feasibility of the health check

#### Participant identification and recruitment process

A sub-set of autistic adults, carers and supporters, and primary care staff (health professional and administrative staff) in the health check arm will be invited to take part in the qualitative study. Autistic adults, carers and supporters, and primary care staff will consent to take part in the qualitative study as part of the main trial consent process described above. Following health checks, the research team will send an information sheet and consent form to primary care staff involved in delivering the health check via email.

We will conduct interviews with up to 20 purposively sampled autistic adults, up to 20 carers and supporters, and up to 30 primary care staff from the health check arm. We will purposively sample autistic adults based on practice, age, gender, and responses from their health check evaluation questionnaire. We will also aim to purposively sample primary care staff based on role and the practice (rural vs urban, large vs small practices). Once selected, the participant will be invited to take part in the interview via email or letter (in line with participant’s preference) and will receive a copy of the information sheet to read again to remind them of the qualitative activities and how their interview data will be managed. The interviews will be conducted face to face or via video or telephone, in line with participants’ preferences, and will be recorded and transcribed verbatim for qualitative analysis.

#### Interview methods

Semi-structured interviews will be undertaken to gather information about the feasibility and acceptability of the health check, the availability of reasonable adjustments, reasons for non-attendance, and barriers and facilitators to implementation in the NHS. The interviews will be analysed using thematic analysis and informed by implementation science theory, in particular Normalisation Process Theory [40] to focus on what the implementation challenges and mechanisms of embedding health checks into practice are.

#### Data analysis

The interviews will be analysed using an inductive approach with subsequent mapping to Normalisation Process Theory framework (NPT [40]). The components of NPT framework look at coherence (i.e. meaning and sense making), cognitive participation (i.e. commitment and engagement), collection action (i.e. work participants do to make the intervention function), and finally reflexive monitoring (i.e. reflections or appraisals of the intervention) [41]. Throughout the analysis, we will engage with the wider research team and patient and public involvement (PPI) groups to check and discuss analysis and interpretation of the interview data. This approach will ensure the analysis is both rigorous and trustworthy.

### Project management

The study consortium includes experienced autism researchers, health professionals including GPs, autistic adults, trial managers, health economists, statisticians, and representatives of the National Autistic Society. The study consortium will meet every 3 to 4 months for the duration of the study. Cumbria, Northumberland, Tyne, and Wear NHS Foundation Trust will sponsor and monitor the study. We will conduct monitoring visits with sites after the first consent to ensure adherence to consent and data storage procedures and maintenance of investigator site file; this will be carried out for each sites undertaking their own consents and for one randomly selected site in each trial arm where RDN and the research team have completed consent.

### Patient and public involvement

The outline of this study was co-designed with autistic people at a two-day summit on Autism and Ageing: Health and Wellbeing [18]. Autistic people and representatives of the National Autistic Society, as part of the study consortium, will be involved all aspects of the study, including co-designing the methods and study documents, supporting the engagement of autistic people in the development of the health check, and developing and co-delivering training, interpretation of results, and dissemination.

### Dissemination plan

We will disseminate the trial findings in a number of ways. The research team and autistic advisors will create accessible and easily understandable summaries of the research findings for each of the stakeholder groups. We will create a short film to host on our website explaining what the health check involves for autistic people, carers and supports, and research participants from all stages of the research programme. The trial results will be presented at academic meetings and in research papers submitted to open-access, peer-reviewed scientific journals. The research team will create a media plan to communicate key messages and support autistic people and carers and supporters to tell their stories. Dissemination within healthcare services and to professionals will help identify suitable pathways in service provision and use in other services.

At the end of the trial, we will offer practices in the usual care arm training on autism. If we find the health check is effective, the research team will also offer training on health check delivery, make health checks materials available, and provide funding if required to those practices to support them to offer participants the health check.

## Discussion

### Significance

The need to address the barriers for autistic adults accessing primary healthcare is a research and policy priority [42]. This research programme is part of such efforts through the creation and evaluation of a UK Primary Care health check for autistic adults. This trial will test the clinical and cost-effectiveness of the health check, developed in collaboration with key stakeholders, and provide important information about the acceptability and feasibility of delivering the health check in NHS Primary Care. If we find the health check is effective, then primary care professionals could use it widely to overcome barriers to healthcare, and potentially improve health outcomes, for autistic adults. This trial will also provide evidence regarding the adjustments to standard healthcare practices required by autistic people and the implementation of these in NHS Primary Care services to help support autistic people’s healthcare access.

### Strengths and limitations

This trial is evaluating the effectiveness and cost-effectiveness of a health check for autistic adults that has been developed in collaboration with autistic people, carers and supporters, and primary care staff. This trial includes adults with a clinically confirmed diagnosis of autism and therefore potentially excludes adults who have struggled to access or engage with autism assessment services and may self-identify as autistic.

## Trial status

Protocol Version 9.0 (14/03/2024). The first participant was recruited to the RCT on 18 April 2023 and the last participant on 21 March 2024. Primary care staff and carers and supporters were recruited to the qualitative study from 12 September 2023 to 31 July 2024. The trial is monitored by the sponsoring NHS trust. The trial is registered with ISRCTN and the NIHR Portfolio. The trial is due to end on 31 May 2025.

## Trial sponsorship

The trial is sponsored by Cumbria, Northumberland, Tyne, and Wear NHS Foundation Trust. Address: St Nicholas Hospital, Jubilee Road, Gosforth, Newcastle upon Tyne, NE3 3XT. Tel: 0191 2,081,356. Web: www.cntw.nhs.uk

## Supplementary Information

Additional file 1.

Additional file 2. Appendix 1: Consent forms.

## Data Availability

The final trial dataset (anonymised) will initially only be accessible by the Trial Management Team lead by the co-lead investigators (JP and BI). The anonymised datasets generated during and/or analysed during the current study will subsequently be available upon request from the corresponding author. Quantitative and qualitative data will become available from June 2026 until 2034. The data will be shared with academic and health service professionals for pre-specified analyses through a contract with Newcastle University as decided by the chief investigators and Health Checks for Autistic Adults Consortium. If the research data shows acceptability and efficacy, the Health Check for Autistic Adults manual and associated materials will be available from the authors in the future.

## Abbreviations

AASPIRE: Academic-Autism Spectrum Partnership in Research and Education
AHAT: Autism Healthcare Accommodations Tool
BNF: British National Formulary for Medications
CHAP: Comprehensive Health Assessment Program
CEACs: Cost-effectiveness acceptability curves
RDN: Research Delivery Network
ISRCTN: International Standard Randomised Controlled Trial Number
NHS: National Health Service
NICE: National Institute for Health and Care Excellence
NIHR: National Institute for Health and Care Research
NPT: Normalisation Process Theory
PAQ: Pre-Appointment Questionnaire
P-COQ: Primary Care Outcomes Questionnaire
PROMs: Patient-Reported Outcome Measures
QALY: Quality-Adjusted Life Year
REC: Research Ethics Committee
RCT: Randomised controlled trial
SF-36 v2: 36 Item Short-Form Health Survey version 2
SF-6D: Short-Form Six Dimensions
SRS-2: Social Responsiveness Scale-Second Edition
W-ADL: Waisman Activities of Daily Living Scale

## Footnotes

1 We use the term ‘autistic people’ in keeping with previous consultation and published research suggesting that this term is favoured by autistic people.

2 We use the term ‘intellectual disability’ in keeping with international classification systems and research—intellectual disability is often referred to as ‘learning disability’ in the UK NHS.
