# Supplementary material for: Health checks for autistic adults: study protocol for a cluster randomised controlled trial
Source: Trials. 2024 Dec 31;25:858. doi: 10.1186/s13063-024-08641-5 (PMC11686900; doi:10.1186/s13063-024-08641-5)
Supplement: Supplementary file 2 — Additional file 2. Appendix 1: Consent forms. [file 13063_2024_8641_MOESM2_ESM.pdf]

Health Checks for Autistic Adults Study

Participant Consent Form – Health check group

Please read the following statements. If you agree please <u>initial</u> the box.

Please <u>initial</u> boxes

1. I have read and understand the information sheet "Information sheet for autistic adults in the Health Checks Group v3 28.06.2022". I have had the opportunity to consider the information, ask questions and have had any questions answered satisfactorily. ☐

2. I understand that my participation is voluntary and that I am free to stop taking part in the study at any time. I know I don't have to give a reason for stopping and it would not affect the care that I receive. If I stop taking part, I understand that the information collected up to that point will still be included in the study, unless I request for it be removed. ☐

3. I consent to Newcastle University securely holding my identifiable information and the research information I give ('data'). ☐

4. I understand that all identifying information will be kept confidential and stored separately from research information (data). ☐

5. I understand that any identifiable information I provide for the study will remain confidential. I understand that the research team may have to break my confidentiality if they had significant concerns about my safety or someone else's safety. The team will aim to keep me informed during any such procedure. ☐

6. I understand that information from relevant sections of my GP/NHS health records will be collected as part of the study and that this information will be looked at by members of the research team at Newcastle University. ☐

7. I understand that information from the "health check pre-appointment questionnaire" that I complete will be part of the research information that will be ☐

looked at and recorded by the research team at Newcastle University. **(Please note this is optional, you do not have to consent to this if you do not want to. If you decide not to consent, you can still take part in the study).**

8. I understand that information from relevant sections of my GP/NHS health records will be collected 3 years after the health check as part of the study and that this information will be looked at and recorded by members of the research team at Newcastle University. **(Please note this is optional, you do not have to consent to this if you do not want to. If you decide not to consent, you can still take part in the study).** ☐

9. I understand that identifiable information from this study will be looked at by members of the research team at Newcastle University. I understand that my identifiable information may also be looked at by the teams ensuring the quality of the research, including from Cumbria, Northumberland, Tyne and Wear NHS Foundation Trust or their representatives. I give permission for these people to have access to the information that the research team collect. ☐

10. I understand that the results of the study will be published. I will not be identifiable in any numerical analyses or quotes, which will be anonymous. ☐

11. I understand that the information collected will be anonymised and may be used to support other research in the future. ☐

12. I consent to take part in the above study. ☐

______________________	____/____/____	________________________
Print Name	Date	Signature/Mark
______________________	____/____/____	________________________
Name of Researcher	Date	Signed by Researcher

Health Checks for Autistic Adults Study

Participant Consent Form - Treatment as Usual Group

Please read the following statements. If you agree please initial the box.

Please initial boxes

1. I have read and understand the information sheet "Information sheet for autistic adults in the Treatment as Usual Group v3 28.06.2022". I have had the opportunity to consider the information, ask questions and have had any questions answered satisfactorily. ☐

2. I understand that my participation is voluntary and that I am free to stop taking part in the study at any time. I know I don't have to give a reason for stopping and it would not affect the care that I receive. If I stop taking part, I understand that the information collected up to that point will still be included in the study, unless I request for it be removed. ☐

3. I consent to Newcastle University securely holding my identifiable information and the research information I give ('data'). ☐

4. I understand that all identifying information will be kept confidential and stored separately from research information (data). ☐

5. I understand that any identifiable information I provide for the study will remain confidential. I understand that the research team may have to break my confidentiality if they had significant concerns about my safety or someone else's safety. The team will aim to keep me informed during any such procedure. ☐

6. I understand that information from relevant sections of my GP/NHS health records will be collected as part of the study and that this information will be looked at by members of the research team at Newcastle University. ☐

7. I understand that information from relevant sections of my GP/NHS health records will be collected as part of the study 3 years after joining the study and that this information will be looked at and recorded by members of the research team at Newcastle University. **(Please note this is optional, you do not have to consent to this if you do not want to. If you decide not to consent, you can still take part in the study).** ☐

8. I understand that identifiable information from this study will be looked at by members of the research team at Newcastle University. I understand that my identifiable information may also be looked at by the teams ensuring the quality of the research, including from Cumbria, Northumberland, Tyne and Wear NHS Foundation Trust or their representatives. I give permission for these people to have access to the information that the research team collect. ☐

9. I understand that the results of the study will be published. I will not be identifiable in any numerical analyses or quotes, which will be anonymous. ☐

10. I understand that the information collected will be anonymised and may be used to support other research in the future. ☐

11. I consent to take part in the above study. ☐

______________________	____/____/____	________________________
Print Name	Date	Signature/Mark
______________________	____/____/____	________________________
Name of Researcher	Date	Signed by Researcher

Health Checks for Autistic Adults Study

Consultee declaration form for relative or carer on behalf of an adult who is unable to give consent – Health Check Group

Please read the following statements. If you agree please initial the box.

Please initial boxes

1. I have been asked to assent to participate on behalf of an autistic person who is unable to give consent in this research project (an adult who lacks capacity). I have read and understood the information sheet "Information sheet for relative or carer of an adult who is unable to give consent – Health Check Group v4 19.01.2023". I have had the opportunity to consider the information, ask questions and have had any questions answered satisfactorily. ☐

2. I have discussed and explained the research as far as possible with the participant about whom I am giving information. In my opinion, he/she/they would have no objection to taking part in this study ☐

3. I agree to participate on behalf of the autistic person whom I'm representing, and answer questions on their behalf. ☐

4. I understand that my participation is voluntary and that I am free to stop taking part in the study at any time. I know I don't have to give a reason for stopping and it would not affect the care that the person I'm representing receives. If I stop taking part, I understand that the information collected up to that point will still be included in the study, unless I request for it be removed. ☐

5. I consent to Newcastle University securely holding my identifiable information and research information I give ('data'). ☐

6. I consent to Newcastle University securely holding identifiable information and research information I give in relation to the autistic person whom I'm representing.

7. I understand that all identifying information will be kept confidential and stored separately from research information (data). ☐

8. I understand that any identifiable information I provide for the study will remain confidential. I understand that the research team may have to break my confidentiality if they had significant concerns about my safety or someone else's safety. The team will aim to keep me informed during any such procedure. ☐

9. I understand that information from relevant sections of their GP/NHS health records will be collected as part of the study and that this information will be looked at by members of the research team at Newcastle University. ☐

10. I understand that information from the health check pre-appointment questionnaire that I complete on behalf of the autistic person will be part of the research information that will be looked at and recorded by the research team. **(Please note this is optional, you do not have to consent to this if you do not want to. If you decide not to consent, you can still take part in the study).** ☐

11. I understand that information from relevant sections of the autistic person's GP/NHS health records will be collected 3 years after the health check as part of the study and that this information will be looked at and recorded by members of the research team at Newcastle University. **(Please note this is optional, you do not have to consent to this if you do not want to. If you decide not to consent, you can still take part in the study).** ☐

12. I understand that identifiable information from this study will be looked at by members of the research team at Newcastle University. I understand that identifiable information may also be looked at by the agencies ensuring the quality of the research, including from Cumbria, Northumberland, Tyne and Wear NHS Foundation Trust or their representatives. I give permission for these agencies to have access to the information that the research team collect. ☐

13. I understand that the results of the study will be published. The information I give will not be identifiable in any numerical analyses and quotes, which will be anonymous. ☐

14. I understand that the information collected will be anonymised and may be used to support other research in the future. ☐

15. I consent to take part in the above study. ☐

____________________________________ ____________________________

Name of Consultee Signature/Mark

____________________________________ ____/____/____

Relationship to autistic person Date

____________________ ____/____/____ ______________________

Name of Researcher Date Signed by Researcher

Health Checks for Autistic Adults Study

Consultee declaration form for relative or carer on behalf of an adult who is unable to give consent – Treatment as Usual Group

Please read the following statements. If you agree please initial the box.

	Please initial boxes
1. I have been asked to assent to participate on behalf of an autistic person who is unable to give consent in this research project (an adult who lacks capacity). I have read and understood the information sheet "Information sheet for relative or carer of an adult who is unable to give consent – Treatment as Usual Groupv4 19.01.2023". I have had the opportunity to consider the information, ask questions and have had any questions answered satisfactorily.	☐
2. I have discussed and explained the research as far as possible with the participant about whom I am giving information. In my opinion, he/she/they would have no objection to taking part in this study	☐
3. I agree to participate on behalf of the autistic person whom I'm representing, and answer questions on their behalf.	☐
4. I understand that my participation is voluntary and that I am free to stop taking part in the study at any time. I know I don't have to give a reason for stopping and it would not affect the care that the person I'm representing receives. If I stop taking part, I understand that the information collected up to that point will still be included in the study, unless I request for it be removed.	☐
5. I consent to Newcastle University securely holding my identifiable information and research information I give ('data').	☐

6. I consent to Newcastle University securely holding identifiable information and research information I give in relation to the autistic person whom I'm representing. ☐

7. I understand that all identifying information will be kept confidential and stored separately from research information (data). ☐

8. I understand that any identifiable information I provide for the study will remain confidential. I understand that the research team may have to break my confidentiality if they had significant concerns about my safety or someone else's safety. The team will aim to keep me informed during any such procedure. ☐

9. I understand that information from relevant sections of their GP/NHS health records will be collected as part of the study and that this information will be looked at by members of the research team at Newcastle University. ☐

10. I understand that information from relevant sections of the autistic person's GP/NHS health records will be collected as part of the study 3 years after joining the study and that this information will be looked at and recorded by members of the research team at Newcastle University. **(Please note this is optional, you do not have to consent to this if you do not want to. If you decide not to consent, you can still take part in the study).** ☐

11. I understand that identifiable information from this study will be looked at by members of the research team at Newcastle University. I understand that identifiable information may also be looked at by the agencies ensuring the quality of the research, including from Cumbria, Northumberland, Tyne and Wear NHS Foundation ☐

Trust or their representatives. I give permission for these agencies to have access to the information that the research team collect.

12. I understand that the results of the study will be published. The information I give will not be identifiable in any numerical analyses and quotes, which will be anonymous. ☐

13. I understand that the information collected will be anonymised and may be used to support other research in the future. ☐

43. I consent to take part in the above study. ☐

______________________________	______________________________
Name of Consultee	Signature/Mark
______________________________	____/____/____
Relationship to autistic person	Date

____________________	____/____/____	____________________
Name of Researcher	Date	Signed by Researcher
